# Projected Oral Health Outcomes and Costs Associated With Pediatric Medicaid Disenrollment

**DOI:** 10.1001/jamanetworkopen.2026.11457

**Published:** 2026-05-12

**Authors:** Sung Eun Choi, Lisa Simon, Catherine Hayes, William V. Giannobile

**Affiliations:** 1 Department of Oral Health Policy and Epidemiology, Harvard School of Dental Medicine, Boston, Massachusetts; 2 Division of General Internal Medicine, Brigham and Women’s Hospital, Boston, Massachusetts; 3 Department of Oral Medicine, Infection, and Immunity, Harvard School of Dental Medicine, Boston, Massachusetts

## Abstract

**Importance:**

Medicaid provides comprehensive dental coverage to more than 37 million children in the US and plays a central role in reducing barriers to care and improving oral health outcomes. Under the 2025 One Big Beautiful Bill Act (OBBBA), many of these children may be at risk of losing their Medicaid coverage.

**Objective:**

To estimate changes in oral health outcomes and health care costs associated with projected Medicaid coverage loss among US children.

**Design, Setting, and Participants:**

A stochastic decision-analytic microsimulation model was developed to project changes in oral health and economic outcomes associated with Medicaid coverage loss among US children aged 0 to 18 years over a 10-year horizon (2025-2034). Simulated population characteristics were informed by the nationally representative National Health and Nutrition Examination Survey (NHANES) 2013-2018. Sensitivity analyses evaluated uncertainty in policy magnitude and structural assumptions. All analyses were performed from July 25 to March 2, 2025.

**Exposure:**

Projected annual Medicaid disenrollment of 480 000 children under the 2025 OBBBA from 2025 through 2034.

**Main Outcomes and Measures:**

Incremental incident caries events and emergency department visits for nontraumatic dental-related conditions, quality-adjusted life-years (QALYs), and health care costs.

**Results:**

The simulated cohort was based on 11 696 NHANES participants aged younger than 19 years (mean [SD] age, 9.2 [0.1] years; 5778 [weighted percentage, 49.2%] females). Over 10 years, projected coverage loss was associated with 95 799 (95% uncertainty interval [UI], 15 107-171 514) additional incident caries events, 27 084 (95% UI, −41 015 to −12 458) QALYs lost, and $86.5 million (95% UI, $47.1-$125.1 million) in additional health care costs in the base-case scenario. Across alternative policy impact scenarios, incremental caries events ranged from 54 051 (95% UI, −5354 to 111 084) to 161 231 (95% UI, 58 630-261 271), and incremental costs ranged from $47.9 million (95% UI, $17.1-$77.6 million) to $145.8 million (95% UI, $92.5-$193.4 million).

**Conclusions and Relevance:**

In this decision-analytic microsimulation modeling study, projected pediatric Medicaid coverage loss was associated with worsening oral health outcomes and increased health care costs. The magnitude of impact varied with the scale of coverage loss but remained adverse across alternative policy scenarios.

## Key Points

**Question:**

What are the projected oral health and cost outcomes associated with Medicaid coverage loss under the 2025 One Big Beautiful Bill Act among US children?

**Findings:**

In this decision-analytic microsimulation modeling study using data for 11 696 children younger than 19 years from the US National Health and Nutrition Examination Survey, Medicaid coverage loss was associated with an additional 95 799 incident caries events and $86.5 million in health care costs over 10 years. Across alternative policy impact scenarios, incremental caries events ranged from 54 051 to 161 231 and incremental costs ranged from $47.9 million to $145.8 million.

**Meaning:**

Results of this study suggest that Medicaid plays a critical role in children’s access to dental care and oral health and coverage disruptions may increase untreated dental disease and health care costs among children reliant on Medicaid.

## Teaser

This decision analytical model assesses the projected oral health and cost outcomes associated with Medicaid coverage loss under the 2025 One Big Beautiful Bill Act among US children.

## Introduction

Children in the US, particularly those from low-income families, continue to face substantial barriers to accessing dental care.^1^ Nearly 1 in 5 US children aged 5 to 19 years have untreated dental caries, with prevalence substantially higher among children from low-income families and those without dental insurance.^2^ Medicaid plays a critical role in addressing this gap in care by offering comprehensive dental coverage to approximately 37.3 million children and adolescents through the Early and Periodic Screening, Diagnostic, and Treatment benefit.^3,4^ Moreover, Medicaid dental benefits have been associated with reductions in emergency department (ED) visits for nontraumatic dental conditions, averting costly and avoidable ED visits.^5^

A recent simulation modeling study highlighted that insurance coverage in childhood is highly dynamic; an estimated 61% of US children ever enroll in Medicaid, and 42% experience a period of uninsurance by age 18, underscoring the existing fragility of coverage continuity.^6^ Despite its foundational importance, the Medicaid program is being challenged by the 2025 One Big Beautiful Bill Act (OBBBA).^7^ Projections suggest that approximately 480 000 children per year could lose Medicaid coverage between 2025 and 2034 if work-reporting requirements apply only to adults who gained coverage through the Medicaid expansion.^8,9^

Loss of Medicaid coverage may disproportionately affect children from low-income families, potentially widening existing gaps in access to dental care and oral health outcomes.^10^ While a prior simulation study estimated the impact of coverage loss on the health system due to policy-driven Medicaid cuts among US adults,^11^ to our knowledge, no studies to date have examined how Medicaid coverage losses among children would be associated with dental care use, oral health outcomes, and system-level costs over time. We used a stochastic microsimulation model to estimate the association between Medicaid coverage loss and oral health outcomes and associated costs among US children.

## Methods

In this decision-analytic modeling study, we developed and applied a microsimulation model to evaluate how the OBBBA Medicaid coverage reductions would be expected to affect dental care use, oral health outcomes, and associated costs among US children. This study was reported in accordance with the Consolidated Health Economic Evaluation Reporting Standards (CHEERS) reporting guideline.^12^ This study was reviewed by the institutional review board of the Harvard Medical School and was determined to be exempt from the requirement of approval and from informed consent because the study used only deidentified publicly available data.

### Data Sources

Baseline demographic, insurance type, dental care use, and clinical data from oral health examinations were obtained from the National Health and Nutrition Examination Survey (NHANES, 2013-2018), the only national survey in the US that contains clinical oral health examination data rather than self-reported dental outcomes.^13^ NHANES data from 2017 to March 2020 indicate that pediatric dental caries prevalence has remained relatively stable,^14^ supporting the use of NHANES 2013-2018 in this study (eTable 1 in Supplement 1). Survey sample weights were used to correct for differential sampling and nonresponse in NHANES.^15,16^ To capture policy-induced changes in coverage, we used pediatric Medicaid disenrollment projections from Manatt Health, as summarized by the Center for American Progress, which reports expected child coverage loss associated with Medicaid work requirements and related policy changes (eMethods in Supplement 1).^8,9^ Dental-related ED visits and their costs were estimated from published literature.^17,18,19^ Other model input parameters are further detailed in Table 1 and eTable 2 in Supplement 1.

**Table 1.** Model Parameters

Parameter	Base-case value, % or mean (SD)	Range	Distributional assumption	Source
No. of US children enrolled in Medicaid and CHIP^a^	37.4 million	NA	NA	CMS^20^
Population characteristics distribution^a^	eTable 3 in Supplement 1	NA	NA	NHANES 2013-2018
Disease risk				
Baseline dental caries	eTable 4 in Supplement 1	NA	NA	NHANES 2013-2018
Baseline dental care utilization	eTable 5 in Supplement 1	NA	NA	NHANES 2013-2018
All-cause mortality rate	eMethods in Supplement 1	NA	NA	CDC^21^
Risk of dental caries	Calibrated; eFigure in Supplement 1	NA	NA	Model-based estimates
Transition to CHIP	21.0%	10.3-21.0	β	Alker et al^22^; MACPAC^23^
Change in annual dental visit probability with Medicaid coverage loss, pp	16.0	0.0-33.0	Scaled β	Howell and Kenney^24^
Probability of untreated caries in privately insured with caries, %	21.3	15.8-26.8	β	Duffy et al^25^
Odds ratio of untreated caries in publicly insured	1.39	1.05-1.84	Log-normal	Duffy et al^25^
Odds ratio of untreated caries in uninsured	1.88	1.13-3.15	Log-normal	Duffy et al^25^
Probability of tooth abscess for untreated caries, %	32.1	30.0-46.4	β	Azodo et al^26^; Srivastava^27^; Schnabl et al^28^
Probability of tooth loss for untreated caries, %	76.6	66.3-85.5	β	Monte-Santo et al^29^
Probability of ED visits for dental-related condition, %				
Privately insured	0.1	0.10-0.12	β	Morgan et al^19^; Cairns et al^18^; Allareddy et al^17^
Publicly insured	0.49	0.49-0.57	β	Morgan et al^19^; Cairns et al^18^; Allareddy et al^17^
Uninsured	0.67	0.67-0.78	β	Morgan et al^19^; Cairns et al^18^; Allareddy et al^17^
Quality-of-life decrement				
Dental caries	0.010 (0.003)	0.0038-0.019	β	IHME^30^; Kay et al^31^
Tooth abscess	0.069 (0.015)	0.029-0.110	β	Brennan et al^32^
Tooth loss	0.067 (0.013)	0.045-0.095	β	IHME^30,33^
Cost, USD				
Examination	88 (10)	45-145	γ	Atkins et al^34^; Humana^35^; ADA^36^
Dental caries	570 (20)	330-977	γ	Atkins et al^34^; Humana^35^; ADA^36^
Tooth abscess	857 (45)	325-1278	γ	Atkins et al^34^; Humana^35^; ADA^36^
Tooth extraction	229 (30)	97-410	γ	Atkins et al^34^; Humana^35^; ADA^36^
ED visits	992 (130)	400-1500	γ	ADA^37^; Wall et al^38^

Abbreviations: ADA, American Dental Association; CDC, Centers for Disease Control and Prevention; CHIP, Children’s Health Insurance Program; CMS, Centers for Medicaid and Medicare Services; ED, emergency department; IHME, Institute For Health Metrics and Evaluation; MACPAC, Medicaid and CHIP Payment and Access Commission; NHANES, National Health and Nutrition Examination Survey; NA, not applicable; pp, percentage points; USD, US dollars.

^a^ While NHANES-based proportions were used to model national insurance-type distributions and capture population characteristics, we used CMS administrative estimates of the number of children enrolled in Medicaid to translate simulation outcomes into absolute national effect estimates among Medicaid-enrolled children.

### Statistical Analysis

#### Simulation Model

We developed a microsimulation model representing a nationally representative cohort of 100 000 US children aged 0 to 18 years, starting in 2025, to estimate changes in total costs, quality-adjusted life-years (QALYs), ED visits, and incident dental caries events associated with Medicaid coverage loss, accounting for differences in demographics, disease risks, and dental care use (eTable 3 in Supplement 1). Each individual was assigned sociodemographic characteristics based on NHANES distributions: age (2-5, 6-12, 13-18 years), sex, race and ethnicity per the NHANES self-identified survey response to the options: Hispanic (Mexican-American or other Hispanic), non-Hispanic Black, non-Hispanic White, or other (individuals who self-identified as belonging to other races or as multiracial and did not identify as Hispanic), income (<130% of the federal poverty level [FPL], middle [130%-300% of FPL], and high [>300% of FPL]), and insurance (private, public, and uninsured).

The simulation followed up a closed cohort of children aged 0 to 18 years in 2025 through 2034 to estimate the downstream consequences of Medicaid disenrollment during childhood. Simulated follow-up ended when individuals reached age 19, at which point they were no longer eligible for the Medicaid pediatric dental benefit. All-cause mortality was incorporated, allowing individuals to exit the cohort over time.^21^ While NHANES-based proportions were used to characterize population attributes, national Medicaid enrollment estimates were used to scale simulation outputs to absolute effect estimates at the population level.^20^

Annual incident dental caries risk was modeled using a logistic functional form including age, race and ethnicity, income, and insurance, with interaction terms to allow age-specific heterogeneity. Initial coefficient values were informed by regression estimates and subsequently calibrated to reproduce NHANES-observed caries prevalence under status quo conditions (eMethods in Supplement 1). The resulting probability estimates were used as annual individual-level transition risks, and incident events were simulated as binary outcomes accumulated over time. To assess face validity of the model, model-projected caries prevalence was compared with NHANES estimates (dental caries defined as having signs of decay, being filled on the crown or enamel surfaces, or missing due to caries)^39^ (eFigure in Supplement 1).

Among individuals with dental caries, the probability of receiving treatment varied by insurance type.^25^ For those with untreated caries, individuals faced a modeled probability of developing caries-related complications, such as tooth abscess and tooth loss, based on published estimates.^26,27,28,29^ ED utilization was modeled as insurance-specific rates.^17,18,19^

We compared outcomes from policy scenarios against a status quo scenario in which Medicaid eligibility and enrollment levels were held constant at pre-OBBBA levels. The primary policy scenario reflects projected Medicaid coverage loss between 2025 and 2034 under the OBBBA. In the base-case scenario, pediatric Medicaid enrollment was modeled as 480 000 lower in each year of the policy window relative to the status quo, reflecting projected effects of work-reporting requirements applied to expansion adults.^8,9^ This represents a sustained net reduction in Medicaid enrollment levels rather than cumulative annual disenrollment events. Within the closed cohort, the enrollment gap was implemented as a proportional reduction in Medicaid coverage each year, with age-specific disenrollment probabilities rescaled to maintain the intended differential and age distribution (eMethods in Supplement 1). To reflect uncertainty in administrative implementation and procedural disenrollment intensity, we evaluated a low-impact scenario assuming 263 000 fewer children enrolled and a high-impact scenario assuming 803 000 fewer children enrolled, based on prior policy modeling analyses.^9^ Consistent with prior evidence, 21.0% of those individuals losing Medicaid coverage were assumed to transition to the Children’s Health Insurance Program (CHIP),^22,23^ with the remainder becoming uninsured. Because private insurance often does not include dental coverage or provides more limited benefits, transitions to private insurance were not modeled in our base-case explicitly^40^; however, 1-way sensitivity analyses examined up to 20% of children obtaining private dental coverage.

The estimated outcomes of the simulation interventions included cumulative incident dental caries events, cumulative ED visits for dental-related conditions, and incremental QALYs and costs. Costs and QALY estimates were integrated over the simulated period for all simulated individuals from a health care perspective. Costs included diagnostic, treatment of dental caries and caries-related complications (including tooth abscess and tooth extraction), and ED expenditures. Cost inputs were obtained from the American Dental Association, claims data, and a prior cost-effectiveness analysis (Table 1).^34,35,36,37,38^ In the absence of pediatric dental-condition–specific preference-based utility weights, disability weights were used as proxy quality-of-life decrement parameters,^30,31,32,33^ consistent with prior health economic modeling when condition-specific utility data are unavailable.^41,42,43^ Costs were expressed in 2025 US dollars using the Consumer Price Index,^44^ Personal Health Care Dental Service, and Personal Consumption Expenditure,^45^ and costs and QALYs were discounted at 3% annually.

#### Sensitivity and Uncertainty Analysis

We evaluated an alternative structural specification of caries risk. In the base case, caries incidence was specified as a calibrated reduced-form function of insurance status in which we did not simultaneously impose an explicit utilization-mediated pathway to avoid double-counting mechanisms. In sensitivity analyses, we modeled insurance transitions as affecting dental visits,^24^ which, in turn, altered the likelihood of receiving preventive services (eg, fluoride varnish and sealants)^46^ with documented protective effects on caries incidence (eMethods in Supplement 1).^47^

In addition, because pediatric quasi-experimental estimates (studies using natural experiments or policy variation to approximate causal identification) for downstream dental outcomes are limited, we incorporated effect estimates from adult Medicaid expansion studies on ED visits and untreated caries.^48,49^ These estimates were evaluated in structural sensitivity analyses to assess the robustness of projected outcomes under alternative specifications (eMethods in Supplement 1).

One-way sensitivity analyses were conducted across plausible ranges of values for model parameters (eTable 2 in Supplement 1). We performed a probabilistic sensitivity analysis by sampling from the probability distributions of all input parameters to capture uncertainties in our estimates (Table 1). The model was re-run 1000 times via Monte Carlo sampling, generating 95% uncertainty intervals (UIs) according to the reporting guidelines.^50,51^

The eMethods and eTables 1 through 5 in Supplement 1 provide all input data and complete technical details. All analyses were performed in 2025 using R, version 4.4.1 (The R Foundation for Statistical Computing).

## Results

**Figure 1.**
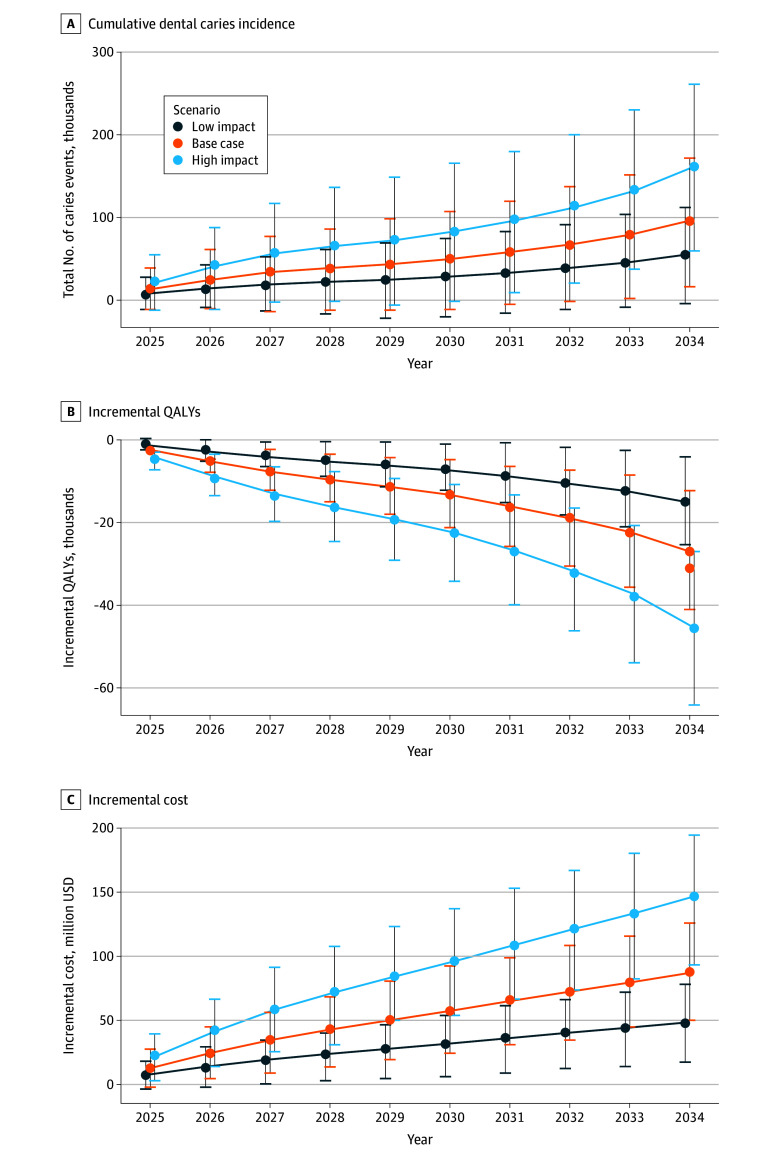
Line Graphs Showing Simulated Outcomes Over a 10-Year Period Results are presented as incremental changes in projected outcomes for each simulated scenario (base case and high impact) compared with the status quo. Results were obtained from 1000 iterations with Monte Carlo sampling, generating 95% uncertainty intervals from the simulation model. QALY indicates quality-adjusted life-year; USD, US dollars.

The simulated population was informed by NHANES data of 11 696 participants (mean [SD] age, 9.2 [0.1] years; 5778 [weighted percentage, 49.2%] females; 3624 [weighted percentage, 24.5%] Hispanic, 2702 [weighted percentage,13.6%] non-Hispanic Black, 3418 [weighted percentage, 51.0%] non-Hispanic White, and 1952 [weighted percentage, 10.9%] other race individuals). If there were no changes to the current Medicaid policy and health risk factor profiles, our model estimated that the dental caries prevalence would be 15.3% (95% UI, 14.6%-16.2%) among individuals aged 2 to 5 years, 48.6% (95% UI, 47.5%-49.6%) among individuals aged 6 to 12 years, and 57.4% (95% UI, 56.2%-58.5%) among individuals aged 13 to 18 years (eFigure in Supplement 1). Additional model calibration results demonstrated that model-projected status quo outcomes matched observed data within 5% absolute error (eFigure in Supplement 1). Distributional plausibility checks further supported the face validity and internal consistency of the model (eTable 6 in Supplement 1). Simulated outcomes over a 10-year period are depicted in Figure 1.

All projected intervention effect estimates are reported as incremental differences relative to the status quo scenario. National estimates were scaled to reflect the projected number of children experiencing Medicaid disenrollment nationally under each policy scenario. Under the base-case scenario of Medicaid coverage loss (480 000 fewer children enrolled) under the OBBBA, the model projected 95 799 (95% UI, 15 107-171 514) additional incident caries events and 7367 (95% UI, −6220 to 19 550) additional ED visits for nontraumatic dental conditions over a 10-year period (Table 2). This corresponded to 0.21 (95% UI, 0.03-0.37) additional caries events and $187.6 (95% UI, $102.0-$271.2) in incremental health care costs per affected child over 10 years, representing a 3.9% increase in incident caries and a 10.6% increase in dental-related health care costs relative to the status quo among affected children (Table 3). At the national level, the base-case scenario was associated with $86.5 million (95% UI, $47.1-$125.1 million) in incremental health care costs and a loss of 27 084 (95% UI, −41 015 to −12 458) QALYs. Across alternative policy impact scenarios, projected 10-year incremental caries events ranged from 54 051 (95% UI, –5354 to 111 084) to 161 231 (95% UI, 58 630-261 271), incremental costs ranged from $47.9 million (95% UI, $17.1-$77.6 million) to $145.8 million (95% UI, $92.5-$193.4 million), and QALY losses ranged from 15 143 (95% UI, −25 711 to −4328) to 45 410 (95% UI, −63 965 to −26 709). These findings quantify the projected oral health and economic consequences of reduced publicly financed pediatric dental coverage under varying levels of coverage loss.

**Table 2.** Projected 10-Year Impact Level of Medicaid Coverage Loss by Scenario (2025-2034)^a^

Scenario (totals only)	Estimate (95% UI)^b^			
	Incident caries events	ED visits for NTDC	QALYs	Costs, million USD
Low impact	54 051 (−5354 to 111 084)	4359 (−4443 to 14 218)	−15 143 (−25 711 to −4328)	47.9 (17.1 to 77.6)
Base case	95 799 (15 107 to 171 514)	7367 (−6220 to 19 550)	−27 084 (−41 015 to −12 458)	86.5 (47.1 to 125.1)
High impact	161 231 (58 630 to 261 271)	13 648 (−1800 to 31 103)	−45 410 (−63 965 to −26 709)	145.8 (92.5 to 193.4)

Abbreviations: ED, emergency department; NTDC, nontraumatic dental-related condition; QALY, quality-adjusted life-year; UI, uncertainty interval; USD, US dollars.

^a^ Total values represent 10-year national projections scaled from the simulated cohort to the US Medicaid-enrolled child population. All values represent incremental changes relative to the status quo scenario over the 10-year period (2025-2034). The base-case scenario assumes 480 000 Medicaid-enrolled children losing coverage over the 2025-2034 policy window under expansion-only work requirements. The low- and high-impact scenarios assume 263 000 and 803 000 children losing coverage, respectively.

^b^ Estimates are based on 1000 Monte Carlo iterations and are presented with 95% UIs. UIs may include zero for some outcomes due to parameter uncertainty. Undiscounted results are provided in eTable 9 in Supplement 1.

**Table 3.** Projected 10-Year Impact of Medicaid Coverage Loss Per Affected Child (2025-2034)^a^

Child impact	Estimate (95% UI)	
	Per affected child	Relative change among affected children, %
Incident caries events	0.21 (0.03 to 0.37)	3.9 (0.6 to 7.1)
ED visits for NTDCs	0.02 (−0.01 to 0.04)	39.0 (−21.9 to 125.0)^b^
QALYs	−0.06 (−0.09 to −0.03)	−0.7 (−1.1 to −0.3)
Costs, USD	187.6 (102.0 to 271.2)	10.6 (5.7 to 15.7)

Abbreviations: ED, emergency department; NTDC, nontraumatic dental-related condition; QALY, quality-adjusted life-year; USD, US dollars.

^a^ All values represent incremental changes relative to the status quo scenario over the 10-year period (2025-2034). Estimates are based on 1000 Monte Carlo iterations and are presented with 95% uncertainty intervals. Uncertainty intervals may include zero for some outcomes due to parameter uncertainty. Per-affected child estimates represent the cumulative 10-year incremental change for a child experiencing coverage loss under the policy scenario compared with their status quo counterfactual. Relative changes among affected children are calculated as percentage differences compared with their projected status quo totals over the same 10-year period.

^b^ Relative percentage changes in ED visits were large due to low baseline rates and wider uncertainty intervals.

Under the alternative utilization-mediated structural specification, projected incremental costs over 10 years were $80.5 million (95% UI, $43.7-$117.9 million), compared with $86.5 million in the base case (eTable 7 in Supplement 1). Incremental QALY losses were 24 255 (95% UI, −38 165 to −10 422), compared with a loss of 27 084 QALYs in the base case. These results were modestly attenuated but remained directionally consistent with the primary estimates.

When applying the quasi-experimental causal estimate for ED visits, projected incremental ED visits were 1697 (95% UI, −8464 to 12 019). Corresponding incremental costs were $82.2 million (95% UI, $56.1-$123.6 million) compared with $86.5 million in the base case. Applying the quasi-experimental causal estimate for untreated caries yielded larger projected effect estimates, including a loss of 34 424 QALYs (95% UI, −50 063 to −20 712) and $115.6 million (95% UI, $75.7-$155.1 million) in additional health care costs, exceeding base-case projections.

**Figure 2.**
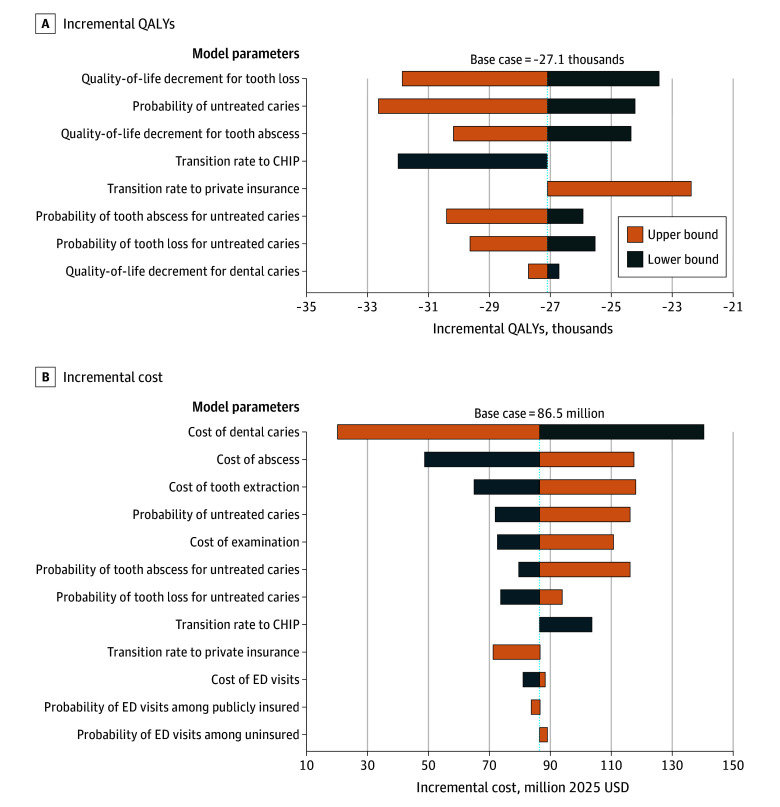
Tornado Diagrams of 1-Way Sensitivity Analysis Results on Incremental QALYs and Cost Results were obtained from 1000 iterations with Monte Carlo sampling from the simulation model. CHIP indicates Children’s Health Insurance Program; ED, emergency department; QALY, quality-adjusted life-year; USD, US dollars.

None of the 1-way sensitivity analyses substantially changed our primary findings, except for the cost of treating dental caries (Figure 2; eTable 8 in Supplement 1). Uncertainty around the cost of treating dental caries was the most influential parameter affecting incremental cost estimates. When the cost of treatment was set at its upper bound, the projected incremental cost associated with Medicaid coverage loss appeared lower (Figure 2). This counterintuitive result reflects the lower likelihood of receiving treatment among uninsured children; as coverage loss shifts children from Medicaid to uninsured status, fewer caries are treated, attenuating the observed increase in health care spending despite higher unit costs. Across all scenarios, reduced access to care among uninsured children was consistently associated with worse health outcomes, as reflected in greater QALY losses.

## Discussion

In this microsimulation modeling study of US children, projected Medicaid coverage loss under the 2025 OBBBA was associated with adverse oral health and economic consequences over a 10-year period. In the base-case scenario, coverage loss was projected to result in 95 799 additional incident caries events and $86.5 million in incremental health care costs. Although estimates for ED visits were associated with wide UIs, findings consistently indicated worsening oral health outcomes and increased system-level costs. While these incremental costs represent a small fraction of total US health expenditures, they reflect preventable disease burden concentrated among socioeconomically vulnerable children, for whom even modest coverage disruptions may have disproportionate clinical and functional consequences. These findings underscore the central role of Medicaid in supporting preventive dental care and limiting downstream disease burden among children.

The projected increases in incident dental caries events and associated health care costs highlight the role of Medicaid dental coverage in facilitating access to preventive and routine oral health care for children. Importantly, projected cost increases should be interpreted as reflecting redistribution within the health care system rather than uniform increases in Medicaid spending. Although a reduction in Medicaid direct dental expenditures may occur under coverage loss, increases in uninsured children may delay or forgo preventive care, resulting in higher rates of untreated disease, caries-related complications, and ED use. These changes may increase total system-level costs with financial burdens shifting to hospitals, safety-net systems, and families. Beyond aggregate expenditure, increased untreated dental disease may strain tertiary care settings, where severe odontogenic infections are associated with substantial uncompensated care. Clinically, untreated caries can result in dental pain, infection, and functional impairment, with documented associations between oral health and school absenteeism.^52^ Although rare, severe odontogenic infections can become life-threatening if untreated and may require hospitalization.^53^ These findings suggest that even moderate coverage disruptions may have disproportionate impacts on socioeconomically vulnerable children.

Children who lose Medicaid coverage under the OBBBA may face limited and uncertain pathways to regain comprehensive dental coverage, increasing the likelihood of prolonged gaps in care. Although some may transition to the CHIP, such transitions are unlikely to fully offset projected coverage losses.^22,23^ State-level variation in CHIP eligibility thresholds, enrollment procedures, benefit design, and administrative capacity may further limit coverage uptake.^54^ In addition, although Medicaid guarantees comprehensive pediatric dental benefits, CHIP dental coverage may be more limited in some states, particularly for restorative procedures, endodontic care, prosthodontic services, and dental-related anesthesia.^55^ Thus, even children who transition to CHIP may experience disruptions in care continuity, reduced access to preventive and restorative services, or higher out-of-pocket costs. These factors suggest that coverage loss may translate into sustained periods of uninsurance or diminished access to comprehensive dental care.

### Limitations

This study has limitations inherent to simulation modeling based on secondary data sources. First, the model focuses on pediatric oral health outcomes and health care costs and does not account for broader societal costs, such as productivity losses due to missed school or parental work absences.^56^ In addition, we did not project the long-term consequences of reduced dental access in childhood on adult outcomes, despite evidence that poor oral health in youth has lasting implications.^56,57^ Therefore, projections should be interpreted as estimates of oral health–specific consequences rather than comprehensive assessments of all downstream effects of coverage loss.

Although the model assumed that children transitioning to the CHIP would receive the same level of dental services as those covered by Medicaid, CHIP dental benefits may be less comprehensive in some states, potentially affecting access to care. We also did not model dynamic policy responses, such as state efforts to expand CHIP eligibility, implement continuous eligibility policies, or increase support for safety-net dental providers, which could mitigate projected impacts.

ED visits were modeled using insurance-specific utilization rates, such that projected changes reflect population-level shifts in insurance composition rather than individual-level causal effects of Medicaid disenrollment. While cross-sectional differences in utilization by insurance status may reflect unobserved confounding, this approach is consistent with observed utilization patterns and is appropriate for estimating population-level burden under alternative coverage scenarios. Additionally, although the model operates at the individual level, age-specific incidence estimates were used to approximate transitions across dentitions, which may obscure tooth-level dynamics but are appropriate for population-level policy projections.

This analysis relied on disability weights as proxies for health-related quality-of-life decrements in the absence of pediatric dental–specific preference-based utility weights, a pragmatic approach used in health economic modeling when condition-specific utility data are unavailable.^41,42^ Because pediatric dental conditions contribute negligible mortality, a disability-adjusted life-year (DALY) framework would largely reflect years lived with disability and would not materially alter interpretation.

More broadly, although our model incorporates extensive data from nationally representative surveys and peer-reviewed literature, all simulation models rely on assumptions and input parameters that may not fully capture the complexity of policy implementation in practice. Some parameters, such as insurance-specific treatment rates, may not fully reflect recent secular changes in practice patterns. We also did not model potential supply-side responses to changes in Medicaid enrollment that could influence access for children who remain enrolled in Medicaid.

Finally, the study used a closed cohort model to represent pediatric Medicaid beneficiaries exposed to annual disenrollment through 2034. Although annual enrollment reductions were calibrated to preserve national magnitude and age distribution, the model does not incorporate new pediatric entrants after baseline. Thus, projected effect estimates reflect sustained policy exposure applied to a representative baseline cohort rather than explicitly modeling population turnover. External validation of long-term projections was not feasible due to the lack of nationally representative longitudinal data on pediatric dental caries outcomes. Instead, the model was calibrated to observed NHANES distributions under status quo conditions, and extensive sensitivity analyses were conducted. As with all decision analytical models, results remain subject to structural assumptions and parameter uncertainty.

## Conclusions

In this simulation modeling study, large-scale Medicaid coverage loss under the 2025 OBBBA was projected to be associated with increased dental caries, higher health care costs, and declines in QALYs among low-income children. These findings highlight the central role of Medicaid in supporting pediatric oral health and suggest that disruptions in coverage continuity may have measurable clinical and economic consequences.

## Notes

Supplement 1. **eMethods.** Model simulation **eTable 1.** Comparison between NHANES 2013-2018 vs 2017- March 2020 **eTable 2.** Model parameters for sensitivity analysis **eTable 3.** Demographic distribution by insurance type **eTable 4.** Baseline prevalence of tooth decay **eTable 5.** Baseline dental utilization **eTable 6.** Distribution of simulated outcomes under the status quo **eTable 7.** Projected 10-Year Impact of Medicaid Coverage Loss (2025–2034) under alternative structural specification: Utilization-mediated caries pathway **eTable 8.** One-way sensitivity analysis results **eTable 9.** Undiscounted projected 10-year impact of Medicaid coverage loss (2025–2034) **eFigure.** Simulation model calibration

Supplement 2.
Data Sharing Statement
