# Supplementary material for: Projected Oral Health Outcomes and Costs Associated With Pediatric Medicaid Disenrollment
Source: JAMA Netw Open. 2026 May 12;9(5):e2611457. doi: 10.1001/jamanetworkopen.2026.11457 (PMC13169400; doi:10.1001/jamanetworkopen.2026.11457)
Supplement: Supplement 1. — eMethods. Model simulation eTable 1. Comparison between NHANES 2013-2018 vs 2017- March 2020 eTable 2. Model parameters for sensitivity analysis eTable 3. Demographic distribution by insurance type eTable 4. Baseline prevalence of tooth decay eTable 5. Baseline dental utilization eTable 6. Distribution of simulated outcomes under the status quo eTable 7. Projected 10-Year Impact of Medicaid Coverage Loss (2025–2034) under alternative structural specification: Utilization-mediated caries pathway eTable 8. One-way sensitivity analysis results eTable 9. Undiscounted projected 10-year impact of Medicaid coverage loss (2025–2034) eFigure. Simulation model calibration [file jamanetwopen-e2611457-s001.pdf]

Supplemental Online Content

Choi SE, Simon L, Hayes C, Giannobile WV. Projected effects of pediatric medicaid disenrollment on oral health outcomes and costs. *JAMA Netw Open*. 2026;9(5):e2611457. doi:10.1001/jamanetworkopen.2026.11457

eMethods. Model simulation

eTable 1. Comparison between NHANES 2013-2018 vs 2017- March 2020

eTable 2. Model parameters for sensitivity analysis

eTable 3. Demographic distribution by insurance type

eTable 4. Baseline prevalence of tooth decay

eTable 5. Baseline dental utilization

eTable 6. Distribution of simulated outcomes under the status quo

eTable 7. Projected 10-Year Impact of Medicaid Coverage Loss (2025–2034) under alternative structural specification: Utilization-mediated caries pathway

eTable 8. One-way sensitivity analysis results

eTable 9. Undiscounted projected 10-year impact of Medicaid coverage loss (2025–2034)

eFigure. Simulation model calibration

This supplemental material has been provided by the authors to give readers additional information about their work.

© 2026 Choi SE et al. *JAMA Network Open*.

eMethods. Model Simulation

Model specification

We developed an individual-level microsimulation model to reproduce current patterns of dental disease, utilization, and insurance coverage among US children and to project oral health and cost outcomes under alternative Medicaid coverage scenarios. The model is stochastic, incorporating parameter uncertainty through Monte Carlo sampling, and operates in annual time steps over a 10-year horizon beginning in 2025.

A nationally representative synthetic cohort of 100,000 US children aged 0 -18 years in 2025 was constructed using NHANES demographic distributions and complex survey weights. Each simulated individual was assigned a combination of age (2-5, 6-12, 13-18 years old), sex, race/ethnicity [National Health and Nutrition Examination Survey (NHANES) categories of non-Hispanic white, non-Hispanic black, Hispanic (Mexican-American or other), and other race], and income [relative to the FPL, adjusted for household size; <130% of the federal poverty level [FPL]), middle (130%-300% of FPL), and high (>300% of FPL)], and insurance types (private, public, and uninsured) (eTable 2). The model was re-run 1000 times while repeatedly Monte Carlo sampling from the probability distributions of model input parameters to capture uncertainties in our estimates.[1] Baseline dental utilization (annual dental visit) and the risk of tooth decay were modeled and assigned to each simulated individual (eTables 3 and 4).

Annual incident dental caries risk was specified using a logistic functional form including age category, race/ethnicity, household income, and insurance type, with interaction terms to allow age-specific heterogeneity in insurance and racial effects. The annual transition probability for individual i in year t was given by:

$$p_{i,t} = \frac{\exp(\eta_{i,t})}{1 + \exp(\eta_{i,t})}$$

Where $\eta_{i,t}$ is a linear predictor including demographic and insurance covariates and interaction terms. Initial coefficient values were informed by logistic regression models estimated from nationally representative survey data. To ensure consistency with observed disease patterns, all coefficients were subsequently calibrated to reproduce NHANES-observed caries prevalence across age, race/ethnicity, and insurance strata under status quo conditions. Calibration was performed by iteratively adjusting coefficients to minimize the sum of squared deviations between simulated and observed prevalence across strata. Calibration was considered

© 2026 Choi SE et al. *JAMA Network Open*.

achieved when simulated prevalence differed from NHANES estimates by less than 5% (eFigure 1). Dietary behaviors were assumed to remain constant across all policy scenarios, as the simulated insurance transitions were not expected to directly alter baseline dietary habits; therefore, dietary factors were not explicitly modeled to maintain focus on the incremental effects of coverage loss. Annual incident caries events were simulated as Bernoulli draws among individuals, and cumulative caries incidence was obtained by summing events over time. Treatment and complication probabilities were applied conditionally based on disease status and insurance type.

The baseline (status quo) annual dental visit probabilities were modeled using a logistic specification including age, sex, income, and insurance type. In the base-case scenario, changes in visit probability associated with insurance transitions were parameterized using quasi-experimental estimates from Howell et al.,[2] which report a 16 percentage-point increase in annual dental visits associated with Medicaid coverage gains. Coverage loss was modeled as the reverse of this effect (-16 percentage points). To preserve individual heterogeneity in care-seeking behavior over time, each simulated individual retained their percentile rank within the insurance-specific utilization probability distribution. When insurance status changed, the individual's new utilization probability was obtained by mapping their retained percentile to the corresponding quantile of the destination insurance-specific distribution. This approach preserves within-person relative propensity for care use while allowing mean utilization to vary across insurance types.

Among individuals with dental caries, treatment probabilities varied by insurance type.[3] For those with untreated caries, individuals faced modeled probability of developing caries-related complications, such as tooth abscess and tooth loss, based on published estimates.[4-7] Emergency department utilization was modeled as insurance-status–specific rates.[8-10] Under alternative policy scenarios, projected changes reflect shifts in insurance composition and corresponding utilization patterns. Quasi-experimental estimates were incorporated in sensitivity analyses to assess the robustness of results to causal effect assumptions.

The model used a closed cohort design, following children aged 0-18 years in 2025 to estimate the downstream consequences of Medicaid disenrollment during childhood. Simulated follow-up ended when individuals reached age 19, at which point they were no longer eligible for Medicaid's pediatric dental benefit. All-cause mortality was incorporated, allowing individuals to

© 2026 Choi SE et al. *JAMA Network Open*.

exit the cohort over time.[11] We used validated equations of age- and sex-specific risk of all-cause mortality as below,[11]

x = age in years

$$\text{Male: } y = 7e - 6 * e^{0.0773x}$$
$$\text{Female: } y = 4e - 6 * e^{0.0825x}$$

Medicaid coverage change

Pediatric Medicaid disenrollment estimates were based on projections from Manatt Health, as summarized by the Center for American Progress, which estimate substantial child coverage reductions under Medicaid work-reporting requirements applied to adults enrolled through the ACA expansion pathway.[12,13] These projections reflect expected differences in Medicaid enrollment levels rather than cumulative annual disenrollment events.

The simulation follows a closed cohort of children aged 0-18 in 2025 through 2034 without adding new entrants after baseline. To represent the projected policy impact within this design, we implemented coverage changes as a sustained annual net reduction in pediatric Medicaid enrollment relative to the status quo during the 2025–2034 period.

Because the number of Medicaid-covered children in the closed cohort declines over time due to aging out and mortality, annual disenrollment probabilities were dynamically rescaled to maintain the intended enrollment differential. Specifically, transition probabilities were multiplied by the ratio, $\frac{N_0}{N_t}$, where N_0 denotes the number of Medicaid-covered children at baseline and N_t is the number of at-risk children in year t. This scaling preserves the projected annual enrollment gap within the shrinking at-risk population.

To maintain representativeness of the pediatric population over time, coverage changes were assigned using age-stratified probabilities calibrated to preserve the baseline pediatric Medicaid age distribution across simulation years.

In the base-case scenario, pediatric Medicaid enrollment was modeled as 480,000 lower in each year of the policy window relative to the status quo, reflecting projected effects of work-reporting requirements applied to expansion adults. This represents a sustained net reduction in enrollment levels rather than cumulative annual disenrollment events.

© 2026 Choi SE et al. *JAMA Network Open*.

To reflect uncertainty in administrative implementation and procedural disenrollment intensity, we evaluated lower- and higher-impact scenarios assuming annual net enrollment reductions of 263,000 and 803,000 children, respectively, based on alternative implementation scenarios described in Manatt Health.[12,13]

Consistent with prior evidence on Medicaid–CHIP transitions, we assumed that 21.0% of children losing Medicaid coverage transition to the Children's Health Insurance Program (CHIP), with the remainder becoming uninsured.[14] Although a small proportion may transition to employer-sponsored or Marketplace plans following Medicaid disenrollment, private insurance often does not include dental coverage or provides more limited benefits; therefore, transitions to private insurance were not modeled explicitly.[15]

<u>Sensitivity Analyses</u>

Alternative Structural Specification: Utilization-Mediated Caries Pathway: In the base-case model, caries incidence was specified as a calibrated reduced-form function of insurance status and demographic characteristics. This specification captures the overall association between insurance coverage and caries risk observed in nationally representative data.

To evaluate whether results were sensitive to structural assumptions regarding the pathway linking insurance to oral health outcomes, we implemented an alternative utilization-mediated specification. Under this approach, insurance transitions first affected annual dental visit probability, parameterized using quasi-experimental estimates from Howell et al.[2] Preventive services (e.g., topical fluoride varnish and sealants) were modeled as occurring during dental visits. Based on national estimates indicating that approximately 48% of Medicaid-enrolled children receive preventive dental services annually,[16] we rescaled this proportion to reflect preventive receipt conditional on having a dental visit in a given year (eTable 2). Receipt of preventive care was modeled as reducing annual caries incidence by a multiplicative relative risk of 0.78 derived from published literature.[17]

In this specification, the direct insurance coefficient in the caries incidence equation was removed to avoid double counting mechanisms. Instead, insurance affected caries risk indirectly through its impact on dental utilization and preventive service receipt. All other model components were unchanged.

© 2026 Choi SE et al. *JAMA Network Open*.

Causal-effect estimates from expansion studies: Because pediatric quasi-experimental estimates for dental outcomes are limited, we incorporated causal evidence from Medicaid expansion studies conducted in settings with comprehensive dental benefits. For ED visits, we applied the difference-in-differences estimate from Giannouchos et al.,[18] which found an 11.4 per 100,000 quarterly reduction in dental-related ED visits following Medicaid expansion. This corresponds to an annual risk difference of 45.6 per 100,000 persons. We applied the reverse of this estimate under coverage loss by increasing annual ED visit probability among affected children by the corresponding risk difference relative to their status quo counterfactual, thereby replacing the insurance-composition–based ED mechanism in the base-case model. For untreated caries, we used estimates from Elani et al.,[19] which found a 16.8 percentage-point reduction in untreated decayed teeth prevalence following Medicaid expansion with dental benefits. In a separate structural sensitivity analysis, we applied the reverse of this estimate under coverage loss by increasing the probability of untreated caries among affected children relative to their counterfactual status quo probability. These alternative specifications allow us to assess the robustness of projected outcomes under causal assumptions derived from quasi-experimental evidence.

Cost and QALYs

Costs and QALY estimates were integrated over the simulated period for all simulated individuals from a healthcare perspective. Costs included those associated with dental diagnostic services, treatment of dental caries, treatment of caries-related complications (including tooth abscess and tooth extraction), and ED visits. Preventive dental services were not modeled as a separate cost category; instead, reduced preventive care among uninsured children was reflected indirectly through lower treatment uptake and higher rates of untreated disease and downstream complications. Cost inputs were obtained from the American Dental Association, claims data and a prior cost-effectiveness analysis (Table 1).[20-24] Health-related quality-of-life decrements were incorporated to estimate QALY losses. In the absence of pediatric dental health state–specific utility weights derived from preference-based instruments, disability weights from large-scale survey data and prior cost-effectiveness analyses,[25-28] were used as proxy quality-of-life decrement parameters, a pragmatic approach that used in health economic modeling when condition-specific utility data are unavailable.[29,30] Disability weights were applied as approximations of utility loss (utility ≈ 1 − disability weight), consistent with prior health economic modeling when condition-specific preference-based utility data are unavailable.[29,30] These proxy weights were applied at the individual level. A caries-related

© 2026 Choi SE et al. *JAMA Network Open*.

disutility was assigned when a child experienced one or more active carious lesions during a model cycle, rather than being applied per affected tooth, to avoid assuming linear aggregation of disutility across multiple affected teeth. Costs were expressed in 2025 US Dollars using the Consumer Price Index,[31] Personal Health Care Dental Service, and Personal Consumption Expenditure,[32] and costs and QALYs were discounted at 3% annually.

© 2026 Choi SE et al. *JAMA Network Open*.

eTable 1. Comparison between NHANES 2013-2018 vs 2017- March 2020: dental caries prevalence, mean (SE)

Although nationally representative dental examination data necessarily lag the simulation start year, the most recent federal oral health surveillance suggests no substantial change in pediatric dental caries prevalence through the most recent available period.

Specifically, CDC analyses of NHANES oral health surveillance data through 2017–March 2020 indicate that approximately 46% of US children aged 2–19 years had experienced dental caries, with persistent levels of untreated decay and stable disparities by socioeconomic status and insurance coverage.[33] These estimates are comparable in magnitude to those observed in earlier NHANES cycles, including 2013–2018, and do not suggest a major secular shift in pediatric caries prevalence over this period.

Our analysis focuses on incremental changes in outcomes attributable to Medicaid coverage loss, rather than forecasting absolute caries prevalence. Under this framework, the use of NHANES 2013–2018 to characterize baseline population risk would be appropriate as long as relative differences in disease burden and care access by insurance status remain broadly stable, which is supported by the most recent surveillance data below.

		NAHNES 2013-2018	NHANES 201-March 2020
Age	<6	0.156 (0.010)	0.149 (0.011)
	6-12	0.481 (0.016)	0.478 (0.027)
	13-18	0.547 (0.013)	0.536 (0.021)
Sex	Male	0.418 (0.012)	0.410 (0.018)
	Female	0.406 (0.012)	0.388 (0.019)
Race	Hispanic	0.496 (0.016)	0.469 (0.020)
	White	0.364 (0.012)	0.363 (0.017)
	Black	0.406 (0.020)	0.406 (0.031)
	Other	0.401 (0.019)	0.403 (0.023)
Insurance	Private	0.356 (0.014)	0.349 (0.021)
	Public	0.453 (0.012)	0.446 (0.014)
	Uninsured	0.491 (0.080)	0.478 (0.045)

© 2026 Choi SE et al. *JAMA Network Open*.

eTable 2. Model parameters for base-case and sensitivity analyses

Parameter	Base-case value % or mean (SD)	Ranges	Distributional assumption	Sources
Transition to CHIP	21.0%	10.3-20.1	Beta	[34,35]
Change in annual dental visit probability with Medicaid coverage loss (percentage points)	16.0 pp	0.0-33.0	Scaled Beta	[2]
Probability of untreated caries in privately insured	21.3%	15.8-26.8%	Beta	[3]
Odds ratio of untreated caries in publicly insured	1.39	1.05-1.84	Normal	[3]
Odds ratio of untreated caries in uninsured	1.88	1.13-3.15	Normal	[3]
Probability of tooth abscess for untreated caries	32.1%	30.0-46.4%	Beta	[4,5,7]
Probability of tooth loss for untreated caries	76.6%	66.3-85.5%	Beta	[6]
Probability of ED visits for dental-related condition*				
Privately insured	0.1%	0.10-0.12%	Beta	[8-10]
Publicly insured	0.49%	0.49-0.57%	Beta	[8-10]
Uninsured	0.67%	0.67-0.78%	Beta	[8-10]
Quality-of-life decrement				
Dental caries**	0.010 (0.003)	0.0038-0.019	Beta	[27,36]
Tooth abscess	0.069 (0.015)	0.029–0.110	Beta	[25]
Tooth loss	0.067(0.013)	0.045–0.095	Beta	[28,36]
Cost, USD				
Examination	88(10)	45-145	Gamma	[20,37,38]
Dental caries	530 (20)	325-977	Gamma	[20,37,38]
Tooth abscess	818 (45)	309-1220	Gamma	[20,37,38]
Tooth extraction	181 (10)	96-360	Gamma	[20,37,38]
ED visits	992 (130)	400-1500	Gamma	[23,24]
Structural Sensitivity Analyses				
Effect of insurance change on ED visits for dental conditions (per 100,000)	45.6	71.6-19.6	Normal	[18]
Effect of insurance change on untreated dental caries (percentage point)	16.8	25.5-8.0	Scaled Beta	[19]
Probability of receiving preventive care - fluoride varnish and sealant	0.48	0.32-0.62	Beta	[16]
Effectiveness of preventive care	0.78	0.65-0.94	Beta	[17]

© 2026 Choi SE et al. *JAMA Network Open*.

*Emergency department visit rates by insurance types

Base-case values: Based on Cairns et al.,[9] (2020 data), overall ED visit rate was 31/100. Total children population size in 2020 was 73.1 million, which results in 22661000 ED visits. Based on Morgan et al.,[8] 89.3 per 10,000 ED visits were related to NTDC.

NTDC ED visits = 89.3/10000 pediatric dental visits = 22661000 * 89.3/10000 = 202362
NTDC ED visit rates = 202362/73.1 million 0.27% of the overall children.
Of NTDC ED visits, 20.1% were private, 65.3% were Medicaid, 12.0% were uninsured, 2.6% were other.[8]

202362*20.1% = 40674
202362*65.3% = 132142
202362*12.0% = 24283

Based on https://www.childstats.gov/americaschildren/tables/hc1.asp, children insurance type distribution in 2020 was 55.1% private, 36.5% Medicaid, 5% uninsured. By applying these insurance type distributions to the overall children, out of total of 73.1 mil children, 40.3 million were private, 26.7 million were public, and 3.65 million were uninsured.
Using these estimates, NTDC ED visits rates by insurance types among us children are,

40674/40.3mil = 0.10%
132142/26.7mil = 0.49%
24283/3.65mil = 0.665%

Upper bound values: Based on Morgan et al.,[8] 0.323% of children (73.6 million children in 2017) had 237728 ED visits for non-traumatic dental related (NTDC) conditions.
Of NTDC ED visits, 20.1% were private, 65.3% were Medicaid, 12.0% were uninsured, 2.6% were other.

Private: 237728*20.1% = 47783
Public: 237728*65.3% = 155236
Uninsured: 237728*12.0% = 28527

Based on https://www.childstats.gov/americaschildren/tables/hc1.asp, children insurance type distribution in 201 was 55.2% private, 36.7% Medicaid, 5% uninsured. By applying these insurance type distributions to the overall children, out of total 73.6 million children, 40.6 million were private, 27.0 million were public, and 3.68 million were uninsured.

Using these estimates, NTDC ED visits rates by insurance types among us children are,

47783/40.6million = 0.12%
155236/27.0million = 0.57%
28527/3.68 million = 0.78%

**For dental caries disutility weight, base-case value is from the Global Burdens of Disease (GBD).[36] The upper bound of the range came from the upper bound of the disability weights in the GBD study and the lower bound came from Kay et al[27]. Kay et al proposed using acute otitis media (a middle ear infection which also involves acute pain and hospital admissions) as an approximation to calculate the impact of tooth decay when it causes pain, due to the lack of utility estimates for the impact of dental caries from the literature. There were three utility

© 2026 Choi SE et al. *JAMA Network Open*.

estimates for otitis media (OM): 0.72, 0.79, 0.882 in Kay et al[27]. We used the highest utility weight of 0.882 (corresponding to the lowest disutility weight) in Kay et al[27] to calculate the lower bound for the disutility weight for dental caries. The steps to calculate the lower bound for the child with caries is as follows:

- Utility weight of extraction (estimated from OM): 0.882
- Duration of disutility: 12 weeks
- QALY loss for extraction: (1-0. 882)*(12/52) [difference between disutility of decayed and unerupted tooth, multiplied by the time for which pain/extraction impacted]
- Children with caries who experience acute pain: 13.91%
- Mean QALY loss per child with caries: (1-0. 882)*(12/52) *.1391= 0.0038

© 2026 Choi SE et al. *JAMA Network Open*.

eTable 3. Demographic distribution by insurance type (weighted proportion)

	Private<br>N=4686<br>(0.51)	Public<br>N=6101<br>(0.43)	Uninsured<br>N=800<br>(0.06)
Dental caries	0.356	0.453	0.491
Age			
0 to 5	0.263	0.353	0.257
6 to 12	0.380	0.374	0.315
13 to 18	0.357	0.273	0.428
Sex			
Male	0.497	0.519	0.536
Female	0.503	0.481	0.464
Race/ethnicity			
Hispanic	0.134	0.347	0.423
NH White	0.664	0.349	0.393
NH Black	0.082	0.204	0.100
Other	0.120	0.101	0.084
Income			
<=130% FPL	0.095	0.652	0.490
130-300% FPL	0.291	0.279	0.380
>300% FPL	0.614	0.069	0.130

Estimates obtained from NHANES – column-wise distribution

© 2026 Choi SE et al. *JAMA Network Open*.

eTable 4. Baseline prevalence of tooth decay

			<6	<6	6-12	6-12	13-18	13-18
Sex	Race/ethnicity	Insurance	Mean	SE	Mean	SE	Mean	SE
Male	Hispanic	Private	0.13	0.03	0.45	0.04	0.53	0.06
		Public	0.20	0.03	0.67	0.03	0.67	0.04
		Uninsured	0.44	0.11	0.70	0.07	0.68	0.07
	NH White	Private	0.10	0.02	0.40	0.03	0.50	0.04
		Public	0.15	0.03	0.53	0.04	0.52	0.05
		Uninsured	0.29	0.10	0.57	0.10	0.70	0.09
	NH Black	Private	0.12	0.04	0.39	0.05	0.40	0.06
		Public	0.20	0.03	0.55	0.03	0.57	0.05
		Uninsured	0.15	0.14	0.27	0.10	0.50	0.12
	Other	Private	0.14	0.03	0.49	0.03	0.38	0.06
		Public	0.21	0.06	0.57	0.06	0.51	0.07
		Uninsured	0.10	0.07	0.40	0.14	0.46	0.11
Female	Hispanic	Private	0.14	0.03	0.39	0.05	0.51	0.04
		Public	0.31	0.03	0.71	0.03	0.70	0.03
		Uninsured	0.31	0.09	0.53	0.08	0.69	0.05
	NH White	Private	0.07	0.01	0.37	0.03	0.52	0.04
		Public	0.13	0.02	0.46	0.04	0.60	0.04
		Uninsured	0.07	0.05	0.29	0.08	0.45	0.12
	NH Black	Private	0.18	0.05	0.36	0.05	0.47	0.07
		Public	0.16	0.02	0.56	0.04	0.57	0.03
		Uninsured	0.12	0.08	0.37	0.11	0.65	0.14
	Other	Private	0.14	0.04	0.42	0.04	0.56	0.07
		Public	0.23	0.03	0.60	0.07	0.57	0.05
		Uninsured	0.19	0.12	0.59	0.18	0.75	0.11

Estimates obtained from NHANES

© 2026 Choi SE et al. *JAMA Network Open*.

eTable 5. Baseline Dental Utilization

			<6	<6	6-12	6-12	13-18	13-18
Sex	Race/ethnicity	Insurance	Mean	SE	Mean	SE	Mean	SE
Male	Hispanic	Private	0.58	0.07	0.87	0.03	0.86	0.04
		Public	0.68	0.03	0.93	0.02	0.76	0.03
		Uninsured	0.57	0.10	0.77	0.04	0.43	0.07
	NH White	Private	0.56	0.03	0.91	0.02	0.84	0.03
		Public	0.60	0.04	0.81	0.04	0.82	0.05
		Uninsured	0.23	0.09	0.45	0.11	0.49	0.12
	NH Black	Private	0.60	0.06	0.86	0.04	0.86	0.04
		Public	0.61	0.03	0.84	0.02	0.77	0.03
		Uninsured	0.50	0.18	0.54	0.12	0.44	0.09
	Other	Private	0.53	0.04	0.88	0.02	0.88	0.03
		Public	0.53	0.05	0.75	0.05	0.68	0.10
		Uninsured	0.34	0.14	0.54	0.18	0.62	0.15
Female	Hispanic	Private	0.55	0.06	0.87	0.04	0.82	0.04
		Public	0.74	0.03	0.92	0.02	0.83	0.02
		Uninsured	0.49	0.09	0.70	0.06	0.53	0.05
	NH White	Private	0.60	0.04	0.92	0.02	0.92	0.02
		Public	0.58	0.04	0.89	0.03	0.69	0.06
		Uninsured	0.25	0.10	0.72	0.10	0.78	0.08
	NH Black	Private	0.64	0.05	0.89	0.03	0.87	0.02
		Public	0.70	0.03	0.88	0.02	0.84	0.04
		Uninsured	0.59	0.19	0.69	0.13	0.58	0.17
	Other	Private	0.49	0.06	0.89	0.03	0.87	0.05
		Public	0.61	0.06	0.88	0.03	0.72	0.06
		Uninsured	0.32	0.15	0.79	0.14	0.84	0.07

Estimates obtained from NHANES

© 2026 Choi SE et al. *JAMA Network Open*.

eTable 6. Distribution of simulated outcomes under the status quo (10-year horizon)

As an internal consistency check, we examined the distribution of cumulative incident dental caries and ED visits under the status quo scenario. Across simulation runs, the mean cumulative incidence per individual ranged from 5.16 to 5.62 over the 10-year horizon, indicating a narrow and plausible distribution without implausible extremes.

Statistic	Cumulative dental caries (N per person)	Cumulative ED visits (N per person)
Min	5.158	0.0192
25th percentile	5.328	0.0404
Median	5.376	0.0462
Mean	5.377	0.0475
75th percentile	5.424	0.0539
Max	5.617	0.0963

© 2026 Choi SE et al. *JAMA Network Open*.

eTable 7. Projected 10-Year Impact of Medicaid Coverage Loss (2025–2034) under alternative structural specification: Utilization-mediated caries pathway

A. Per-Affected Child Impact (10-year cumulative)		
	Per-affected child	Relative change among affected (%)
Incident caries event	0.19 (0.02, 0.35)	3.6 (0.4, 6.6)
ED visits for NTDC	0.02 (-0.01, 0.04)	39.0 (-21.9, 125.0)
QALYs	-0.05 (-0.08, -0.02)	-0.6 (-1.0, -0.3)
Costs	174.5 (94.8, 255.5)	9.9 (5.3, 14.7)

B. National Impact by Scenario (Totals Only)				
	Incident caries events (thousands)	ED visits for NTDC (thousands)	QALYs (thousands)	Costs (USD, million)
Low impact	47.8 (-10.4, 107.0)	4.6 (-4.4, 14.2)	-13.3 (-24.3, -2.8)	44.7 (15.8, 71.8)
Base case	87.8 (10.5, 162.4)	7.9 (-5.3, 20.4)	-24.3 (-38.2, -10.4)	80.5 (43.7, 117.9)
High impact	149.7 (42.4, 245.1)	13.3 (-3.6, 30.2)	-41.2 (-58.8, -23.1)	135.8 (83.8, 185.4)

© 2026 Choi SE et al. *JAMA Network Open*.

eTable 8. One-way sensitivity analysis results

Parameter	Lower Bound	Incremental QALYs	Incremental Cost	Upper Bound	Incremental QALYs	Incremental Cost
Transition rate to CHIP	0.103	-32.0 (-46.7, -19.9)	103.3 (64.4, 151.9)	0.210	-27.1 (-41.5, -13.0)	86.5 (16.4, 155.0)
Transition rate to private	0.000	-27.1 (-41.5, -13.0)	86.5 (16.6, 155.3)	0.200	-22.4 (-33.4, -10.8)	71.2 (34.5, 105.9)
Probability of untreated caries in privately insured	0.16	-24.2 (-37.6, -12.8)	72.0 (42.0, 100.7)	0.27	-32.6 (-45.3, -19.6)	104.9 (66.5, 142.4)
Probability of tooth abscess for untreated caries	0.30	-25.9 (-37.5, -12.4)	79.5 (9.1, 148.6)	0.46	-30.4 (-43.0, -16.7)	116.2 (74.4, 154.2)
Probability of tooth loss for untreated caries	0.66	-25.5 (-37.1, -12.2)	73.7 (1.8, 142.9)	0.86	-29.6 (-42.6, -16.2)	94.0 (61.1, 125.7)
Probability of ED visits among privately insured	0.0010	x	86.5 (16.6, 155.3)	0.0012	x	86.5 (16.4, 155.0)
Probability of ED visits among publicly insured	0.0049	x	86.5 (16.6, 155.3)	0.0057	x	83.6 (17.1, 155.8)
Probability of ED visits among uninsured	0.0067	x	86.5 (16.6, 155.3)	0.0078	x	89.1 (18.9, 158.7)
Cost						
Examination	45.00	x	72.6 (42.6, 102.8)	185.00	x	110.8 (76.2, 144.5)
Dental caries	330.00	x	140.4 (86.2, 218.1)	977.00	x	20.1 (-70.9, 98.0)
Tooth abscess	325.00	x	48.7 (22.8, 67.4)	1278.00	x	117.5 (73.8, 157.9)
Tooth extraction	97.00	x	65.0 (33.7, 94.1)	410.00	x	118.0 (81.7, 155.4)
ED visits	400.00	x	81.1 (16.3, 151.7)	1500.00	x	88.3 (19.7, 162.0)
Disutility weight						
Dental caries	0.0038	-26.7 (15.0, 134.1)	x	0.019	-27.6 (-39.6, -13.9)	x
Tooth abscess	0.0290	-24.3 (-36.2, -11.3)	x	0.110	-31.2 (-44.3, -17.4)	x
Tooth loss	0.0450	-23.4 (-35.3, -10.8)	x	0.095	-31.8 (-43.8, -17.8)	x

© 2026 Choi SE et al. *JAMA Network Open*.

eTable 9. Undiscounted projected 10-year impact of Medicaid coverage loss (2025–2034)

B. National Impact by Scenario (Totals Only)				
	Incident caries events (thousands)	ED visits for NTDC (thousands)	QALYs (thousands)	Costs (USD, million)
Low impact	54.1 (-5.3, 111.1)	4.4 (-4.4, 14.2)	-17.5 (-29.9, -5.0)	55.7 (19.9, 90.2)
Base case	95.8 (15.1, 171.5)	7.4 (-6.2, 19.6)	-31.5 (-47.6, -14.5)	100.5 (54.7, 145.4)
High impact	161.2 (58.6, 261.3)	13.46 (-1.8, 31.1)	-52.8 (-74.4, -31.0)	169.4 (107.5, 224.7)

eFigure. Simulation model calibration

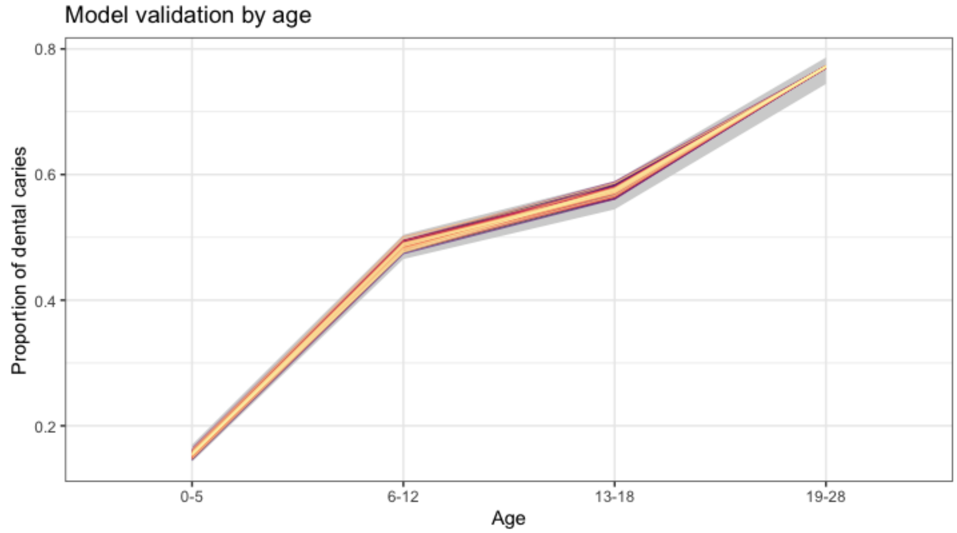

By insurance type

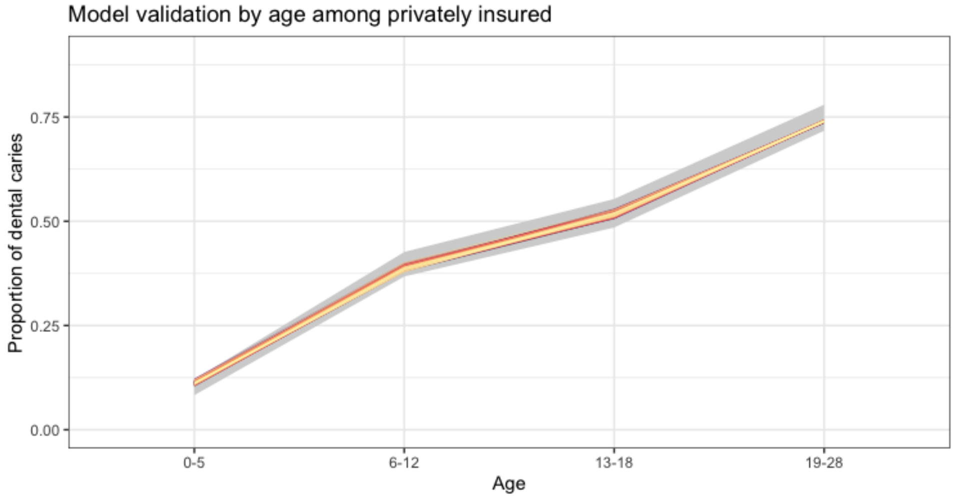

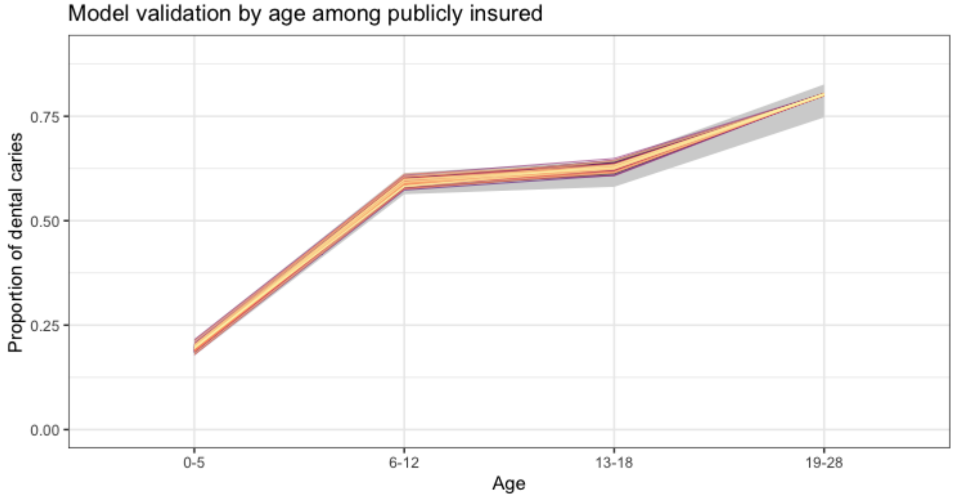

Model validation by age among uninsured

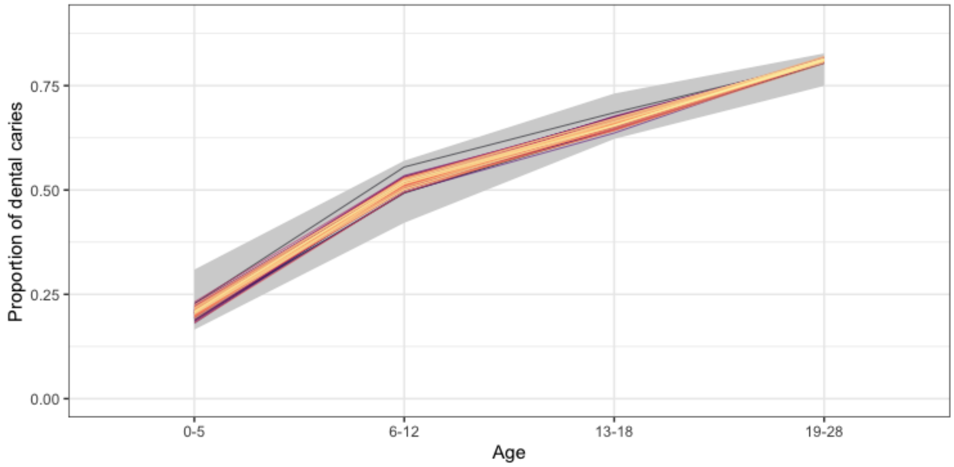

By Race

Model validation by age among Hispanic

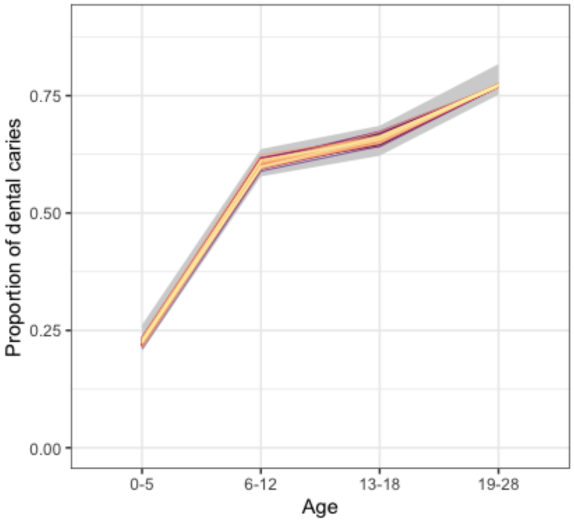

Model validation by age among White

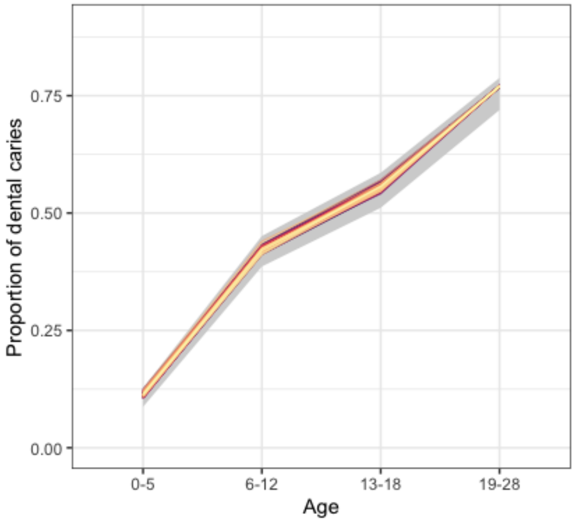

Model validation by age among Black

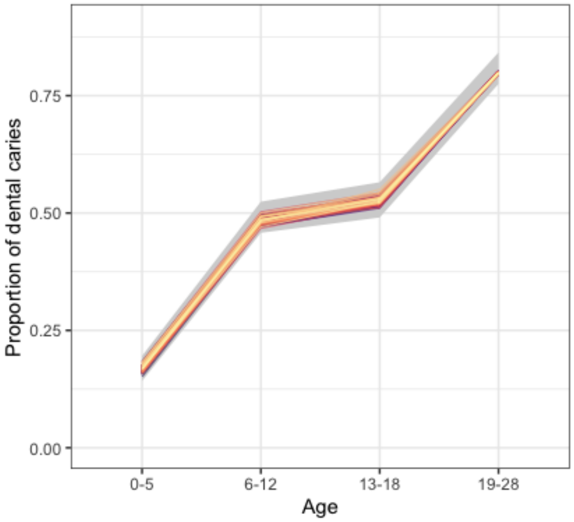

Model validation by age among Other

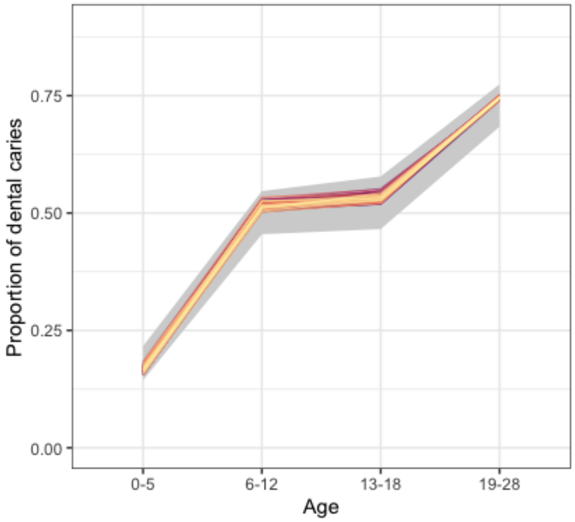

References

1. Ramsey S, Willke R, Briggs A, et al. Good research practices for cost-effectiveness analysis alongside clinical trials: the ISPOR RCT-CEA Task Force report. *Value Health*. Sep-Oct 2005;8(5):521-33. doi:10.1111/j.1524-4733.2005.00045.x
2. Howell EM, Kenney GM. The impact of the Medicaid/CHIP expansions on children: a synthesis of the evidence. *Med Care Res Rev*. Aug 2012;69(4):372-96. doi:10.1177/1077558712437245
3. Duffy EL, Kranz AM, Dick AW, Sorbero M, Stein BD. Association between type of health insurance and children's oral health, NHANES 2011-2014. *J Public Health Dent*. Sep 2018;78(4):337-345. doi:10.1111/jphd.12278
4. Azodo CC, Chukwumah NM, Ezeja EB. Dentoalveolar abscess among children attending a dental clinic in Nigeria. *Odontostomatol Trop*. Sep 2012;35(139):41-6.
5. Srivastava VK. Prevalence of Abscesses Associated with Carious Primary Teeth in Preschool Children and its Association with Age, Gender, Location, and Parent's Education and Social Class: An Observational Study. *Int J Clin Pediatr Dent*. May-Jun 2022;15(3):287-292. doi:10.5005/jp-journals-10005-2376
6. Monte-Santo AS, Viana SVC, Moreira KMS, Imparato JCP, Mendes FM, Bonini G. Prevalence of early loss of primary molar and its impact in schoolchildren's quality of life. *Int J Paediatr Dent*. Nov 2018;28(6):595-601. doi:10.1111/ipd.12416
7. Schnabl D, Fleischer F, Riedmann M, Laimer J, Gassner R. Prevalence and distribution of deep caries and abscess formation in children who required emergency dental general anaesthesia. A retrospective analysis. *Eur J Paediatr Dent*. Jun 2019;20(2):119-122. doi:10.23804/ejpd.2019.20.02.07
8. Morgan T, Samtani MT, Yeroshalmi F, Tranby E, Laniado N, Okunseri C, Badner V. National Trends and Characteristics in Emergency Department Visits for Nontraumatic Dental Conditions Among Pediatric Patients. *Pediatr Dent*. May 15 2021;43(3):211-217.
9. Cairns C, Ashman JJ, Peters ZJ. Emergency Department Visits Among Children Aged 0-17 by Selected Characteristics: United States, 2019-2020. *NCHS Data Brief*. Jun 2023;(469):1-8.
10. Allareddy V, Nalliah RP, Haque M, Johnson H, Rampa SB, Lee MK. Hospital-based emergency department visits with dental conditions among children in the United States: nationwide epidemiological data. *Pediatr Dent*. Sep-Oct 2014;36(5):393-9.
11. Centers for Disease Control and Prevention. United States Life Tables, National Center for Health Statistics. Accessed 2025, Nov 11. http://www.cdc.gov/nchs/products/life_tables.htm
12. Murphy ND, A.;. The Collateral Damage of Medicaid Work Requirements. American Progress. Accessed Jul 1, 2025. https://www.americanprogress.org/article/the-collateral-damage-of-medicaid-work-requirements/
13. Mann CS, K.; Eder, J.; Polk, E.; Tranchina, M.T.; . No Place to Hide: Children Will Be Hurt by Medicaid Cuts. Manatt Health. Accessed Jul 1, 2025. https://assets-us-01.kc-usercontent.com/9fd8e81d-74db-00ef-d0b1-5d17c12fdda9/4e6a5bc9-2dc5-44e5-9760-54833b59d697/LPFCH%20White%20Paper_2025-05_c.pdf
14. Rosenbaum SJ, K.; Bodas, M.; Krips, M.; Jacobs, F. . Deep Medicaid Spending Cuts Put Health Care Coverage at Risk for One of Five Enrolled Children. The Commonwealth Fund Accessed Jul 1, 2025. https://www.commonwealthfund.org/blog/2025/deep-medicaid-spending-cuts-put-health-care-coverage-risk-one-five-enrolled-children
15. Heaton LJOM, J; Burroughs, M.; Santoro, M.; Preston, R.; Tranby, E.P. An Estimated 12 Million Children and Adults Lost Medicaid Dental Insurance. Accessed Aug 10, 2025. https://www.carequest.org/system/files/CareQuest_Institute_An-Estimated-12-Million-Children-and-Adults-Lost-Medicaid-Dental-Insurance_4.1.24_FINAL.pdf

16. Centers for Medicare & Medicaid Services (CMS). CMS Oral Health Initiative and Dental Technical Support Opportunity. Accessed Jan 5, 2026. https://www.medicaid.gov/federal-policy-guidance/downloads/cib062520.pdf#:~:text=SUBJECT:%20CMS%20Oral%20Health%20Initiative%20and%20Dental,52%20percent%20has%20not%20yet%20been%20met.
17. Choi SE, Pandya A, White J, Mertz E, Normand SL. Quality Measure Adherence and Oral Health Outcomes in Children. *JAMA Netw Open*. Jan 2 2024;7(1):e2353861. doi:10.1001/jamanetworkopen.2023.53861
18. Giannouchos TV, Reynolds J, Damiano P, Wright B. Association of Medicaid expansion with dental emergency department visits overall and by states' Medicaid dental benefits provision. *BMC Health Serv Res*. Jun 13 2023;23(1):625. doi:10.1186/s12913-023-09488-3
19. Elani HW, Kawachi I, Sommers BD. Dental Outcomes After Medicaid Insurance Coverage Expansion Under the Affordable Care Act. *JAMA Netw Open*. Sep 1 2021;4(9):e2124144. doi:10.1001/jamanetworkopen.2021.24144
20. Atkins CY, Thomas TK, Lenaker D, Day GM, Hennessy TW, Meltzer MI. Cost-effectiveness of preventing dental caries and full mouth dental reconstructions among Alaska Native children in the Yukon-Kuskokwim delta region of Alaska. *J Public Health Dent*. Jun 2016;76(3):228-40. doi:10.1111/jphd.12141
21. Humana. Cost of common dental procedures. Accessed Jan 5, 2025. https://www.humana.com/dental-insurance/dental-resources/cost-of-dental-procedures
22. American Dental Association. *Survey of Dental Fees*. 2018.
23. American Dental Association (ADA). Emergency department referrals. Accessed Jul 5, 2025. https://www.ada.org/resources/community-initiatives/action-for-dental-health/emergency-department-referrals
24. Wall TV, M. Emergency Department Use for Dental Conditions Continues to Increase. American Dental Association (ADA) Accessed Jul 5, 2025.
25. Brennan DS, Spencer AJ. Disability weights for the burden of oral disease in South Australia. *Popul Health Metr*. Sep 3 2004;2(1):7. doi:10.1186/1478-7954-2-7
26. Institute for Health Metrics and Evaluation (IHME). Global Burden of Disease Study 2019 (GBD 2019) Disability Weights. Accessed Nov 15, 2025. https://ghdx.healthdata.org/record/ihme-data/gbd-2019-disability-weights#:~:text=Disability%20weights%2C%20which%20represent%20the,health%20and%201%20equals%20death
27. Kay E, Owen L, Taylor M, Claxton L, Sheppard L. The use of cost-utility analysis for the evaluation of caries prevention: an exploratory case study of two community-based public health interventions in a high-risk population in the UK. *Community Dent Health*. Mar 1 2018;35(1):30-36. doi:10.1922/CDH_4115Owen07
28. Institute for Health Metrics and Evaluation. Oral disorders. Accessed Nov 11, 2024. https://www.healthdata.org/gbd/methods-appendices-2021/oral-disorders
29. Feng X, Kim DD, Cohen JT, Neumann PJ, Ollendorf DA. Using QALYs versus DALYs to measure cost-effectiveness: How much does it matter? *Int J Technol Assess Health Care*. Apr 2020;36(2):96-103. doi:10.1017/S0266462320000124
30. Neppelenbroek NJM, de Wit GA, Dalziel K, Devlin N, Carvalho NI. Use of Utility and Disability Weights in Economic Evaluation of Pediatric Vaccines. *Value Health*. Jul 2023;26(7):1098-1106. doi:10.1016/j.jval.2023.03.005
31. Bureau of Labor Statistics. Consumer Price Index (CPI) Inflation Calculator. Accessed Jan 5, 2016. http://www.bls.gov/data/inflation_calculator.htm
32. Basu A. Estimating Costs and Valuations of Non-Health Benefits in Cost-Effectiveness Analysis. 2016:

33. Stierman B, Afful J, Carroll MD, et al. National Health and Nutrition Examination Survey 2017-March 2020 Prepandemic Data Files-Development of Files and Prevalence Estimates for Selected Health Outcomes. *Natl Health Stat Report*. Jun 14 2021;(158)doi:10.15620/cdc:106273
34. Medicaid and CHIP Payment and Access Commission (MACPAC). Transitions Between Medicaid, CHIP, and Exchange Coverage. Accessed Jul 1, 2025. https://www.macpac.gov/wp-content/uploads/2022/07/Coverage-transitions-issue-brief.pdf
35. Alker JO, A.; Brooks, T.; Park, E.;. Child Medicaid Disenrollment Data Shows Wide Variation in State Performance as Continuous Coverage Pandemic Protections Lifted. Georgetown University Center for Children and Families. Accessed May 9, 2025. https://ccf.georgetown.edu/2024/05/02/child-medicaid-disenrollment-data-shows-wide-variation-in-state-performance-as-continuous-coverage-pandemic-protections-lifted/
36. Institute for Health Metrics and Evaluation (IHME). Global Burden of Disease Study 2019 (GBD 2019) Disability Weights. Accessed Jan 2, 2022. https://ghdx.healthdata.org/record/ihme-data/gbd-2019-disability-weights#:~:text=Disability%20weights%2C%20which%20represent%20the,health%20and%201%20equals%20death.
37. American Dental Association. Survey of Dental Fees. Accessed Feb 13, 2019. https://success.ada.org/en/practice-management/finances/survey-of-dental-fees
38. Humana. Cost of common dental procedures. Accessed Jan 5, 2022. https://www.humana.com/dental-insurance/dental-resources/cost-of-dental-procedures
