# Supplementary material for: Projected Oral Health Outcomes and Costs Associated With Pediatric Medicaid Disenrollment
Source: JAMA Netw Open. 2026 May 12;9(5):e2611457. doi: 10.1001/jamanetworkopen.2026.11457 (PMC13169400; doi:10.1001/jamanetworkopen.2026.11457)
Supplement: Supplement 2. — Data Sharing Statement [file jamanetwopen-e2611457-s002.pdf]

Data Sharing Statement

Choi. Projected Oral Health Outcomes and Costs Associated With Pediatric Medicaid Disenrollment. *JAMA Netw Open*. Published May 12, 2026.
doi:10.1001/jamanetworkopen.2026.11457

Data
Data available: No

Additional Information
Explanation for why data not available: Data sharing is not applicable to this study as no new data were collected or created.
